# Impact of Biomechanical, Anthropometric, and Temporal Factors on the Return-to-Sport Rate in Recreational Athletes with ACL Reconstruction: A Cross-Sectional Observational Study

**DOI:** 10.3390/healthcare13161970

**Published:** 2025-08-11

**Authors:** Ahmad Alanazi

**Affiliations:** Department of Physical Therapy and Health Rehabilitation, College of Applied Medical Sciences, Majmaah University, Al Majmaah 11952, Saudi Arabia; aalanazi@mu.edu.sa

**Keywords:** anterior cruciate ligament, return-to-sport, biomechanics, rehabilitation, limb symmetry, neuromuscular adaptation

## Abstract

**Background/Objectives**: Anterior cruciate ligament reconstruction (ACLR) necessitates evidence-based rehabilitation strategies to optimize return-to-sport (RTS) outcomes, yet persistent re-injury rates and suboptimal performance persist despite standardized protocols. The purpose of this cross-sectional observational study is to examine the relationship between biomechanical, anthropometric, and temporal factors and return-to-sport outcomes. **Methods**: This cross-sectional study identifies biomechanical, anthropometric, and temporal determinants of RTS readiness in 81 recreational athletes post-ACLR. Outcome measures included anterior (A-SLH), lateral (L-SLH), and medial (M-SLH) single-leg hop for distance, single-leg sit-to-stand (SLSS), single-leg wall-sit hold (SLWS), and ACL-RSI. Statistical analyses employed Spearman’s correlations and multiple linear regression to determine the predictors of ACL-RSI. **Results**: There were significant correlations between RSI and Limb Symmetry Index (LSI) for L-SLH, M-SLH, SLSS, and SLWS (r = 0.27, r = 0.30, r = 0.44, r = 0.34, and *p* < 0.01, respectively). Among the functional outcome measures, multiple linear regression revealed that only SLWS significantly predicted ACL-RSI (β = 0.248, *p* = 0.037). Also, body weight (β = −0.233, *p* = 0.030) and postoperative duration (β = 0.292, *p* = 0.006) significantly predicted ACL-RSI. **Conclusions**: These findings challenge the primacy of limb symmetry indices alone, emphasizing the role of weight management, time-dependent neuromuscular adaptation, and multi-planar closed-chain strength in RTS decision-making. Clinically, rehabilitation frameworks should integrate personalized strategies targeting body composition and dynamic stability to mitigate asymmetric joint loading and enhance functional resilience.

## 1. Introduction

Anterior cruciate ligament (ACL) injuries remain among the most devastating setbacks for athletes, with over 200,000 cases reported annually in the United States, with financial burdens exceeding $7 billion annually in direct or indirect medical costs [1]. ACL injuries are relatively common in Saudi Arabia, but national epidemiological data are scarce. A regional study from Jeddah found that 74 of 282 (26%) knee-injury cases involved an ACL tear [2]. ACL tears are prevalent among adolescents and young adults engaged in high-demand sports such as soccer, basketball, and skiing [3,4]. ACL injury not only disrupts athletic careers but also precipitates long-term health consequences, including early-onset osteoarthritis and chronic joint instability [5]. Despite advances in surgical techniques and rehabilitation protocols, up to 35% of athletes fail to return to their pre-injury performance levels [6], underscoring a critical gap in understanding the multifactorial determinants of sports readiness [7,8]. The ACL Return to Sport Index (ACL-RSI) is a validated patient-reported outcome measure that evaluates an individual’s psychological readiness to return to sport. It comprises 12 items assessing emotions, confidence, and risk appraisal, and is scored on a 0–100 scale, with higher scores indicating greater readiness [9]. Studies have shown that the RSI is a strong predictor of actual return to sport and complements physical performance assessments [10,11]. Previous studies have noted that female athletes tend to report lower ACL-RSI scores compared to males, suggesting heightened fear of re-injury and reduced psychological readiness despite similar physical recovery [12,13]. Differences may reflect variations in emotional response, risk perception, and social pressures influencing return-to-sport decisions.

The Limb Symmetry Index (LSI) is a commonly used measure in return-to-sport assessments following ACL reconstruction. It is calculated as the ratio of performance metrics (e.g., strength, hop distance) on the involved limb to those on the uninvolved limb, multiplied by 100. An LSI ≥ 90% is generally considered acceptable for return-to-sport clearance [14]. However, recent literature suggests that relying solely on LSI may overlook important functional or psychological asymmetries. Central to this challenge is the reliance on RSI, which often prioritizes limb symmetry thresholds (e.g., >85% strength or hop performance between limbs) as benchmarks for relatively safe return to sports [15]. However, athletes who meet these symmetry criteria still face a 15–25% risk of re-injury within two years [16]. For instance, while single-leg hop tests dominate post-ACLR assessments, 72% of non-contact ACL injuries occur during lateral cutting or deceleration movements requiring multiplanar stability rarely captured by mono-planar tasks [17]. This highlights a pressing need to redefine RSI beyond symmetry alone, integrating variables such as body composition, temporal recovery patterns, and foundational strength metrics.

Compounding this issue is the heterogeneity of the ACLR population. Younger athletes (<25 years) often exhibit rapid neuromuscular recovery but heightened psychological distress [18]. A high body mass index (BMI) correlates with 2.3-fold greater odds of asymmetric gait patterns post-surgery, yet weight management remains absent from most rehabilitation frameworks [19,20]. People with ACLR experience clinically significant weight gain during recovery, exacerbating joint loading imbalances and undermining functional progress [21,22]. Such demographic and physiological variability demands a personalized approach to rehabilitation, yet current protocols remain rigidly standardized, often adhering to arbitrary timelines (e.g., 9–12 months post-surgery) rather than individualized adaptation benchmarks [23].

Another important parameter is the role of strength metrics, such as single-leg sit-to-stand (SLSS) and single-leg wall-sit hold (SLWS) tests. While these tests are cornerstones of functional assessments, their predictive utility for RSI remains inconsistent. Research reports a consistent increase in limb symmetry in terms of quadriceps strength from six months to one year [24,25]. Extended postoperative durations may reflect cumulative neuromuscular adaptation rather than passive healing, yet this nuance is rarely operationalized in rehabilitation planning.

To address these gaps, this study investigates the biomechanical, anthropometric, and temporal predictors of ACL-RSI through a hierarchical analytical framework. The purpose of this study was to examine whether the Limb Symmetry Index (LSI) for single-leg hop for distances (SLH) in three directions, single-leg sit-to-stand (SLSS), and single-leg wall-sit hold (SLWS) predict the anterior cruciate ligament-Return to Sport Index (ACL-RSI). It was hypothesized that these functional measures would significantly predict ACL-RSI.

## 2. Materials and Methods

### 2.1. Participants

This cross-sectional observational study was conducted and reported in accordance with the Strengthening the Reporting of Observational Studies in Epidemiology (STROBE) guidelines (Supplementary Table S1). Participants were recruited using a purposive sampling method from individuals undergoing post-ACL reconstruction rehabilitation at the sports rehabilitation centers, orthopedic clinics, and athletic communities in Saudi Arabia. Potential participants were identified through physiotherapy department records and referrals by attending clinicians who were aware of the study inclusion criteria. Eligible participants were identified based on predefined inclusion and exclusion criteria. All participants provided informed consent before enrollment. Efforts were made to ensure voluntary participation, and participants were assured that declining to participate would not affect their care. Eighty-one male athletes (age: 29.9 ± 5.8 years; height: 173.4 ± 5.8 cm; weight: 76.4 ± 15.1 kg; time since ACL reconstruction: 21.8 ± 10.8 months) were recruited. Inclusion criteria required:

1. Unilateral ACL reconstruction with clearance to return to sport from a licensed physiotherapist.
2. Postoperative duration ≥ 9 months (to align with typical rehabilitation timelines for sport-specific readiness [26]).
3. No concurrent lower limb injuries, spinal/pelvic pathologies, or cardiovascular/neurological conditions limiting physical activity.

Exclusion criteria included a history of contralateral ACL injury, recurrent ipsilateral ACL reconstruction, musculoskeletal conditions, neurological disorders, or psychological conditions (e.g., anxiety/depression) affecting ACL-RSI scores [2]. All participants provided written informed consent, and ethical approval was granted by the Institutional Review Board of Majmaah University (approval number: MUREC-DEC.25/COM-2023/36-1), adhering to the Declaration of Helsinki.

### 2.2. Sample Size Calculation

An a priori power analysis was conducted using G*Power software (v3.1.9.7) to determine the minimum sample size required for multiple linear regression analysis. Based on previous studies examining predictors of return-to-sport outcomes [27], a medium effect size (Cohen’s f^2^ = 0.15) was assumed. With eight predictor variables (age, BMI, postoperative duration, hop symmetry in three directions, SLSS time, SLWS time, an alpha level of 0.05, and power of 0.80), the analysis indicated a minimum sample size of 75 participants. To account for potential attrition or incomplete data, the target sample size was set to 85. However, complete data from 81 participants were ultimately analyzed, exceeding the calculated minimum requirement.

### 2.3. Procedures

All participants underwent a single standardized 90-min session at the Physiotherapy and Rehabilitation Laboratory of Majmaah University. The session began with a structured warm-up consisting of 5 min of stationary cycling at 50 watts, followed by dynamic stretching and submaximal hop practice trials.

Immediately after the warm-up, participants completed the Arabic-translated Anterior Cruciate Ligament–Return to Sport after Injury (ACL-RSI) scale to assess psychological readiness. This was followed by a battery of functional performance tests, including the single-leg hop test (anterior, medial, lateral directions), the single-leg sit-to-stand test (SLSS), and the single-leg wall-sit hold test (SLWS). All assessments were conducted in the same order for each participant by trained physiotherapists to ensure protocol consistency.

#### 2.3.1. Psychological Readiness Assessment

The Arabic-translated Anterior Cruciate Ligament–Return to Sport after Injury (ACL-RSI) scale [28] assessed psychological readiness. Participants rated 12 items (e.g., confidence, fear of re-injury) on a 0–10 Likert scale (0 = no confidence, 10 = complete confidence). Total scores were converted to percentages (0–100%), with higher scores indicating greater readiness.

#### 2.3.2. Functional Performance Testing

A standardized warm-up (5-min cycling at 50 W, dynamic stretching, and submaximal hop trials) preceded testing to minimize injury risk.

(a) Single-Leg Hop Tests

Hop symmetry was evaluated in anterior, medial, and lateral directions using validated protocols [29,30]:

- Anterior Hop: Participants hopped forward maximally from a marked line; distance (meters) was measured from the line to the heel.
- Medial/Lateral Hop: Participants hopped sideways (medially or laterally) from a parallel stance; distance was measured from the line to the foot’s lateral/medial edge.
- Symmetry Calculation: the Limb Symmetry Index (LSI) was computed as (Involved limb distance/Uninvolved limb distance) × 100 (Involved limb distance/Uninvolved limb distance) × 100. Three trials per leg were averaged.

(b) Single-Leg Sit-to-Stand Test (SLSS):

Muscular endurance was quantified as the time (seconds) to complete five repetitions unilaterally [31]. Participants sat on a standard chair (height: 46 cm) and performed sit-to-stand motions without arm support.

(c) Single-Leg Wall-Sit Hold Test (SLWS):

Lower extremity endurance was measured as the time (seconds) participants maintained a 90° knee/hip flexion against a wall while lifting the contralateral leg [32].

#### 2.3.3. Data Collection

Demographic (age, height, weight) and surgical (postoperative duration) variables were recorded. Body mass index (BMI) was calculated as weight (kg)/height (m)^2^. Functional scores (hop symmetry, SLSS time, SLWS time) and ACL-RSI scores were compiled into a secure database (Table 1).

#### 2.3.4. Statistical Procedures

Data analysis was conducted using SPSS 20 (IBM Corp., Armonk, NY, USA), with significance set at *α* = 0.05. Descriptive statistics (mean, standard deviation [SD], skewness, kurtosis, minimum, and maximum) were calculated for demographic variables (age, height, weight, time since surgery) and functional performance outcomes (hop tests, squat scores, and return-to-sport after injury (RSI). Assumption of normality was assessed using the Shapiro–Wilk test. Non-parametric Spearman’s rank-order correlations (ρ) were computed to evaluate bivariate associations between variables due to non-normal distributions observed in functional scores (Table 2).

A multiple linear regression model evaluated predictors of RSI, including anthropometric (age, height, weight), surgical (months since surgery), and strength-related variables (lateral/medial hop test, single-leg squat score [SLSS], squat performance symmetry index). Assumptions of independence (Durbin-Watson = 1.748; acceptable range: 1.5–2.5) and multicollinearity (VIFs ≤ 2.489; thresholds < 3.0) were met. Unstandardized (*B*) and standardized coefficients (*β*) with standard errors (SE), 95% confidence intervals (CI), *t*-statistics, and *p*-values were computed. Non-significant predictors (e.g., age, SLSS) were retained to avoid omitted variable bias, following a theory-driven approach. All variables were entered simultaneously, and effect sizes were interpreted via adjusted *R*^2^. No data transformations or imputations were required due to complete data.

## 3. Results

### 3.1. Demographic Data

Eighty-one participants with ACLR participated in this study, with a mean age of 29.94 years (SD = 5.84), height of 173.44 cm (SD = 5.80), and weight of 76.40 kg (SD = 15.13). The mean duration since surgery was 21.84 months (SD = 10.75). Symmetry index scores for the left and right leg hop tests were highest for the anterior direction (M = 90.38, SD = 8.77), followed by lateral (M = 89.94, SD = 8.78) and medial (M = 88.63, SD = 10.77), with all values indicating moderate symmetry (100 = perfect symmetry; lower scores = greater asymmetry). SLSS (M = 84.13, SD = 13.52), squat (M = 83.53, SD = 15.34), and RSI (M = 92.74, SD = 29.17) also demonstrated moderate variability. Most functional scores exhibited negative skewness and moderate to high kurtosis, suggesting non-normal distribution and potential asymmetry in performance outcomes (Table 2).

Spearman’s correlation (n = 81) revealed significant positive associations between RSI and months since surgery (r_s_ = 0.390, *p* < 0.01), lateral (r_s_ = 0.277, *p* < 0.05) and medial (r_s_ = 0.300, *p* < 0.01) hop symmetry, SLSS (r_s_ = 0.441, *p* < 0.01), and SLWS (r_s_ = 0.341, *p* < 0.01), indicating enhanced sports readiness with greater limb symmetry, strength, and postoperative recovery time. A weak age correlation (r_s_ = 0.231, *p* < 0.05) suggested older recreational athletes may experience marginally delayed recovery. Anterior hop symmetry (r_s_ = 0.073, *p* = 0.260) showed no association, highlighting the primacy of medial/lateral stability and squat proficiency over anterior-plane performance in rehabilitation. These findings advocate for targeted neuromuscular training emphasizing dynamic symmetry and unilateral control to optimize post-injury sports reentry (Table 3).

### 3.2. Multiple Linear Regression Analysis

A multiple linear regression analysis examined predictors of RSI, including age, anthropometric, surgical, and strength variables. The model accounted for 25.4% of the variance in RSI (adjusted *R*^2^ = 0.254, *F*(8, 72) = 4.398, *p* < 0.001), with a standard error of 25.19913. Key predictors included weight (*β* = −0.233, *B* = −0.450, 95% CI [−0.856, −0.044], *p* = 0.030), surgery (*β* = 0.292, *B* = 0.793, 95% CI [0.235, 1.351], *p* = 0.006), and SLWS (*β* = 0.248, *B* = 0.472, 95% CI [0.028, 0.915], *p* = 0.037), suggesting that lower body mass, surgical history, and greater squat capacity uniquely influenced RSI. Age (*B* = 0.955, *p* = 0.074) and SLSS (*B* = 0.334, *p* = 0.180) showed non-significant trends, while height, lateral, and medial hop tests had no meaningful impact. Collinearity was acceptable (VIFs ≤ 2.489), and residuals indicated no autocorrelation (Durbin-Watson = 1.748). These results highlight weight management and lower-body strength as modifiable factors for enhancing explosive performance, though unaccounted variables likely contribute to RSI variability (Table 4).

## 4. Discussion

This study explores the biomechanical, temporal, and anthropometric determinants of RSI in post-ACL reconstruction athletes, offering critical insights into the interplay between modifiable and non-modifiable factors. Our regression model explained 25.4% of RSI variance, with weight, postoperative duration, and squat performance emerging as significant predictors. These findings challenge conventional rehabilitation paradigms and underscore the need for personalized, biomechanically grounded approaches to optimize athletic reintegration.

The inverse relationship between body mass and RSI (*B* = −0.450, *p* = 0.030) aligns with the findings of Pappas et al., who reported that increased body weight exacerbates asymmetric joint loading, particularly during dynamic tasks such as cutting and landing [33]. The regression coefficient suggests that for every 5-kg reduction in body mass, RSI scores could theoretically improve by approximately 2.25 units. This observation is consistent with findings by Adouni et al., who reported that excess adiposity in obese populations is associated with prolonged ground reaction forces and compensatory kinematic strategies during dynamic tasks [34]. A systematic review by DiSilvestro et al. reported that individuals with obesity are at increased risk of developing knee osteoarthritis following ACL reconstruction, highlighting the long-term joint health implications of excess body mass in this population [35]. Moreover, a study by Harput et al. reported that higher BMI negatively affected quadriceps strength and recovery outcomes in recreational athletes following ACL reconstruction [36]. Clinicians should consider integrating nutritional counseling and metabolic conditioning to mitigate mass-driven asymmetries, particularly in athletes with a BMI > 25 kg/m^2^.

Time since surgery (*B* = 0.793, *p* = 0.006) emerged as the strongest surgical predictor of RSI, with each additional month post-ACLR correlating with a 0.79-unit readiness improvement. This would imply that with time, post-ACL reconstruction athletes undergo neuromuscular recalibration, and it is not just a passive healing process. Neuroplastic adaptations, including enhanced proprioceptive acuity and intermuscular coordination, likely underpin this trend. This is supported by longitudinal fMRI studies by Grooms et al., which demonstrate cortical reorganization in patients following ACL reconstruction over time [37]. However, our results caution against rigid adherence to 9–12-month timelines. For instance, an athlete 18 months post-surgery with poor squat symmetry may still require delayed clearance despite extended recovery time.

Squat symmetry (*B* = 0.472, *p* = 0.037) outperformed hop tests in predicting RSI, implying that closed-chain strength underpins multi-planar stability. Unlike single-leg hops, squats demand coordinated activation of the quadriceps, hamstrings, and gluteal complexes across multiple planes, similar to sport-specific movements such as deceleration and lateral pivoting [38]. This finding aligns with electromyographic evidence from Ebert et al., who reported greater gluteus medius activation during weighted squats compared to hopping tasks, suggesting exercise-specific muscle recruitment patterns relevant to rehabilitation [39]. Our findings advocate for squat-centric rehabilitation protocols to enhance force absorption capacity and reduce re-injury risk.

While medial (*ρ* = 0.300, *p* < 0.01) and lateral hop symmetry (*ρ* = 0.277, *p* < 0.05) correlated with RSI, their non-significant regression effects (*B* = 0.075 and −0.255, *ps* > 0.59) suggest these metrics could be mediated by squat performance. This may suggest that multiplanar stability during hop tasks may rely on foundational strength developed through squat training—a hypothesis supported by kinetic chain theory and further substantiated by the findings of Almansoof et al., who emphasized the transfer of strength gains from closed-chain exercises to dynamic stability tasks [40]. Conversely, the null effect of anterior hop symmetry (*ρ* = 0.073, *p* > 0.05) underscores its limited ecological validity for sports requiring lateral agility. Clinicians should prioritize medial/lateral hop tests as functional benchmarks but pair them with strength-based interventions to address root causes of asymmetry.

The strong bivariate correlation between SLSS and RSI (*ρ* = 0.441, *p* < 0.01) dissolved in regression analysis (*B* = 0.334, *p* = 0.180), suggesting SLSS effects are confounded by squat capacity. As suggested by Peebles et al., this discrepancy may reflect task specificity: while the single-leg sit-to-stand (SLSS) test evaluates unilateral balance under bodyweight load, squats assess maximal strength, which may serve as a more robust predictor of dynamic stability [41]. Standardizing squat depth and load in future studies could clarify this relationship.

Though age showed a marginal correlation with RSI (*ρ* = 0.231, *p* < 0.05), its non-significant regression effect (*B* = 0.955, *p* = 0.074) aligns with mixed literature on aging and ACLR outcomes. While younger athletes often demonstrate faster strength recovery following ACL reconstruction, Vutescu et al. reported that they also tend to experience higher levels of psychological distress during rehabilitation, potentially affecting their readiness to return to sport [42] and offsetting biomechanical advantages. This underscores the need for holistic assessments integrating mental health metrics.

Clinical Implications

1. Weight Stratification: Embed body composition analytics into rehabilitation, particularly for athletes with BMI > 25 kg/m^2^.
2. Squat-Centric Protocols: Prioritize bilateral squats over isolated hop tests to fortify multiplanar stability.
3. Multiplanar Focus: Integrate lateral/medial hop tests into assessments, but pair them with strength interventions.

Limitations

1. Unexplained Variance: 74.6% of RSI variability remains unaccounted for, possibly due to unmeasured outcome variables such as psychosocial (e.g., kinesiophobia) [43] and genetic factors (e.g., collagen polymorphisms) [44].
2. Observational design of this study precludes causal inference.
3. Sample Homogeneity: Male cohort limits generalizability to female athletes, who face 2–8 times higher ACL injury risk [45].
4. Quadriceps strength was assessed using functional performance tests rather than a standardized clinical grading tool such as the Medical Research Council (MRC) scale. While these tests reflect dynamic strength and sport-specific function, the absence of formal muscle grading may limit the clinical generalizability of strength outcomes. Future studies should consider incorporating both functional and standardized strength assessments.

Future Directions

1. Longitudinal Mixed Models: Track neuromuscular adaptation trajectories across postoperative phases.
2. Psychosocial Integration: Explore interactions between biomechanical readiness and psychological resilience.
3. Biomechanical Profiling: Use 3D motion capture to quantify joint loading asymmetries during squats and hops.
4. Genetic Markers: Investigate collagen gene polymorphisms as moderators of recovery efficiency.

## 5. Conclusions

This cross-sectional study identified body mass, duration since surgery, and functional squat performance as significant predictors of psychological readiness to return to sport following ACL reconstruction. These findings suggest that specific biomechanical and temporal factors are associated with higher ACL-RSI scores. Further prospective studies are needed to validate these associations and explore their role in clinical decision-making.

## Figures and Tables

**Table 1:** Summary of demographic, surgical, psychological, and functional performance variables.

Variable	Category	Type/Scale	Measurement Description
Age	Demographic	Continuous (years)	Self-reported and verified from records
Height	Demographic	Continuous (cm)	Measured using standard stadiometer
Weight	Demographic	Continuous (kg)	Measured using calibrated digital scale
Body Mass Index (BMI)	Derived Demographic	Continuous (kg/m^2^)	Calculated as weight (kg)/height^2^ (m^2^)
Postoperative duration	Surgical	Continuous (months)	Time since ACL reconstruction, verified from surgical records
ACL–Return to Sport Index (RSI)	Psychological	0–100% (Likert-based scale)	Twelve-item Arabic-translated ACL-RSI scale (0–10 per item); total converted to percentage
Single-Leg Anterior Hop Distance	Functional Performance	Continuous (m)	Maximal forward hop from a marked line; average of three trials per leg
Single-Leg Medial/Lateral Hop	Functional Performance	Continuous (m)	Sideways hop distance (medial/lateral); average of three trials per direction per leg
Hop Limb Symmetry Index (LSI)	Functional Symmetry Index	Percentage (%)	(Involved/Uninvolved) × 100; computed for each hop direction
Single-Leg Sit-to-Stand (SLSS)	Functional Endurance	Continuous (s)	Time to complete five reps on each leg without arm support
Single-Leg Wall-Sit Hold Test (SLWS)	Functional Endurance	Continuous (s)	Duration of maintaining 90° wall-sit with opposite leg lifted

**Table 2:** Descriptive statistics for study variables.

	Mean	Std. Deviation	Skewness	Kurtosis	Minimum	Maximum
**Age**	29.94	5.84	0.15	−0.91	18	41
**Height**	173.44	5.8	−0.63	−0.17	159	184
**Weight**	76.4	15.13	0.58	−0.51	51	112
**Surgery**	21.84	10.75	1.8	3.87	9	60
**A-SLH**	90.38	8.77	−1.83	4.15	53.6	99.66
**L-SLH**	89.94	8.78	−1.46	1.94	60.93	99.83
**M-SLH**	88.63	10.77	−1.42	1.78	53.88	99.78
**SLSS**	84.13	13.52	−1.28	0.98	45.88	100
**SLWS**	83.53	15.34	−1.5	2.38	22.03	100
**RSI**	92.74	29.17	−0.78	−0.65	21	120

Note: Age: Age of the participants; Height: Height of the participants (in cm); Weight: Weight of the participants (in kg); Surgery: Months since surgery; A-SLH: Anterior single-leg hop distance; L-SLH: Lateral single-leg hop distance; M-SLH: Medial single-leg hop distance; SLSS: Single-leg sit-to-stand; SLWS: Single-leg wall-sit hold; RSI: Return to sport after injury.

**Table 3:** Spearman’s rho correlation matrix between variables (n = 81).

Variable	Age	Surgery	A-SLH	L-SLH	M-SLH	SLSS	SLWS
**RSI**	0.231 *	0.390 **	0.073	0.277 *	0.300 **	0.441 **	0.341 **

Note: Age: Age of the participants; Height: Height of the participants (in cm); Weight: Weight of the participants (in kg); Surgery: Months since surgery; A-SLH: Anterior single-leg hop distance; L-SLH: Lateral single-leg hop distance; M-SLH: Medial single-leg hop distance; SLSS: Single-leg sit-to-stand; SLWS: Single-leg wall-sit hold; RSI: Return to sports after injury. Scoring for all the variables was calculated as left–right symmetry, with a value closer to 100 indicating more symmetry and better performance. * *p* < 0.05; ** *p* < 0.01.

**Table 4:** Coefficient table.

Predictor	*B* (SE)	95% CI	*β*	t	*p*	Tolerance	VIF
**Constant**	2.766 (95.593)	[−187.796, 193.327]	—	0.029	0.98	—	—
**Age**	0.955 (0.526)	[−0.094, 2.004]	0.191	1.814	0.07	0.841	1.19
**Height**	0.157 (0.537)	[−0.914, 1.227]	0.031	0.292	0.77	0.818	1.22
**Weight**	−0.450 (0.204)	[−0.856, −0.044]	−0.23	−2.21	0.03	0.835	1.2
**Surgery**	0.793 (0.280)	[0.235, 1.351]	0.292	2.831	0.01	0.876	1.14
**L-SLH**	−0.255 (0.474)	[−1.200, 0.690]	−0.08	−0.54	0.59	0.458	2.18
**M-SLH**	0.075 (0.413)	[−0.748, 0.898]	0.028	0.182	0.86	0.402	2.49
**SLSS**	0.334 (0.247)	[−0.158, 0.826]	0.155	1.355	0.18	0.714	1.4
**SLWS**	0.472 (0.222)	[0.028, 0.915]	0.248	2.122	0.04	0.682	1.47

Note. Age: Age of the participants; Height: Height of the participants (in cm); Weight: Weight of the participants (in kg); Surgery: Months since surgery; L-SLH: Lateral single-leg hop distance; M-SLH: Medial single-leg hop distance; SLSS: Single-leg sit-to-stand; SLWS: Single-leg wall-sit hold; RSI: Return to sports after injury, *B* = unstandardized coefficient; *β* = standardized coefficient; SE = standard error; CI = confidence interval; VIF = variance inflation factor. Dependent variable: RSI.

## Data Availability

Data used in this study are available from the corresponding author on reasonable request.

## Supplementary Materials

The following supporting information can be downloaded at: https://www.mdpi.com/article/10.3390/healthcare13161970/s1, Table S1: STROBE Statement—checklist of items that should be included in reports of observational studies.
